# PACS2–TRPV1 axis is required for ER–mitochondrial tethering during ER stress and lung fibrosis

**DOI:** 10.1007/s00018-022-04189-2

**Published:** 2022-02-25

**Authors:** Jessica Knoell, Shashi Chillappagari, Lars Knudsen, Martina Korfei, Ruth Dartsch, Danny Jonigk, Mark P. Kuehnel, Konrad Hoetzenecker, Andreas Guenther, Poornima Mahavadi

**Affiliations:** 1 Department of Internal Medicine, Justus-Liebig University (JLU), Gaffkystraße 11, 35392 Giessen, Germany; 2 Universities of Giessen and Marburg Lung Center (UGMLC), Member of the German Centre for Lung Research (DZL), Giessen, Germany; 3 Department of Biochemistry, Faculty of Medicine, JLU, Giessen, Germany; 4 Institute of Functional and Applied Anatomy, Hannover Medical School, Hannover, Germany; 5 Biomedical Research in Endstage and Obstructive Lung Disease Hannover (BREATH), Member of the German Center for Lung Research (DZL), Hannover, Germany; 6 REBIRTH Cluster of Excellence, Hannover, Germany; 7 Institute of Pathology, Hannover Medical School, Hannover, Germany; 8 Department of Thoracic Surgery, Vienna General Hospital, Vienna, Austria; 9 European IPF/ILD Registry and Biobank, Giessen, Germany; 10 Member of the Cardio-Pulmonary Institute (CPI), JLU, Giessen, Germany; 11 Lung Clinic, Agaplesion Evangelisches Krankenhaus Mittelhessen, Giessen, Germany

**Keywords:** Mitochondrial associated membranes, ER stress, Idiopathic pulmonary fibrosis, Capsaicin, TRPV1

## Abstract

Endoplasmic reticulum (ER) and mitochondria (mito) play a vital role in alveolar type II cell (AEC2) homeostasis and are both stressed in patients with idiopathic pulmonary fibrosis (IPF). Up to now, no data are available with regard to ER–mito cross talk and tethering under conditions of IPF. We here demonstrate that ER–mitochondrial tethering is reduced upon experimental ER stress in vitro and in the IPF AECII ex vivo, and this is—at least in part—due to decreased phosphofurin acidic cluster sorting protein 2 (PACS-2, also called PACS2) protein levels. PACS2 levels are influenced by its interaction with the transient receptor potential cation channel subfamily V member 1 (TRPV1) and can be experimentally modified by the TRPV1-modulating drug capsaicin (CPS). Employing alveolar epithelial cells with overexpression of the terminal ER stress signaling factor Chop or the IPF-associated surfactant protein C mutation (SPC^Δexon4^) in vitro, we observed a restoration of PACS2 levels upon treatment with CPS. Similarly, treatment of precision cut lung slices from IPF patients with CPS ex vivo forwarded similar effects. Importantly, in all models such kind of intervention also greatly reduced the extent of alveolar epithelial apoptosis. We therefore conclude that therapeutic targeting of the PACS2–TRPV1 axis represents an interesting novel, epithelial-protective approach in IPF.

**Supplementary Information:**

The online version contains supplementary material available at 10.1007/s00018-022-04189-2.

## Introduction

Endoplasmic reticulum (ER) and mitochondria are major Ca^2+^ storage organelles. About 20% of mitochondria in a cell are juxtaposed to the ER, forming mitochondria-associated membranes (MAMs) [5]. These dynamic, physical, proteinaceous contacts between ER–mitochondria are required for several physiological functions including Ca^2+^ transfer, lipid homeostasis and functional transfer of metabolites and signaling molecules that are important to govern critical cell fate decisions [33]. Under conditions of ER stress, mitochondria relocate to the perinuclear region to tighten their contact with the ER and show increase in Ca^2+^ uptake, ATP production and oxygen consumption [35]. Upon persistent ER stress, when the unfolded protein response (UPR) fails to resolve such stress, pro-apoptotic signals and apoptotic cell death are triggered, and these mechanisms have been linked to alterations in the MAMs and MAM proteins [10]. Characteristic MAM proteins are inositol 1,4,5-triphosphate receptors (IP_3_R), sigma-1 receptor, calnexin, calreticulin, ERp57 and ERp4. Certain core mitochondrial proteins, namely dynamin-related protein-1 (DRP1) and mitochondrial fission and fusion-regulating proteins, mitofusin 1 and 2 (Mfn-1 and Mfn2), respectively, are also involved in modulating interaction between these two organelles [40]. Another multifaceted sorting protein, phosphofurin acidic cluster sorting protein-2 (PACS2), is also located at the ER–mitochondria interface [39]. Absence of PACS2 has been shown to result in mitochondria-dependent apoptosis by inducing cleavage of BAP31, leading to mitochondrial fragmentation and uncoupling from the ER [37].

Idiopathic pulmonary fibrosis (IPF) is a disease of largely unknown etiology and predominantly affects the aged individual, with a median survival of 2–3 years after diagnosis in untreated patients. Patients display progressive dyspnea, decline in lung function and exercise capacity. Two antifibrotic drugs, pirfenidone and nintedanib (Nin), slow down, but do not stop the disease progression and are authorized for IPF. Histologically, IPF lungs are characterized by the usual interstitial pneumonia (UIP) pattern, with hyperplastic AECII covering fibroblast foci. Although multiple cell types are indicated to contribute to the pathogenesis, chronic injury of AECII is an accepted key event that triggers the disease process in IPF [2]. In this regard, we and others reported extensive ER stress signature molecules in the AECII of sporadic IPF patients and that induction of the terminal ER stress pro-apoptotic transcription factor Chop is sufficient enough to drive lung epithelial cell apoptosis and pro-fibrotic signaling [20]. Supporting this, in familial IPF cases with mutations in surfactant protein C (SPC) gene, which lead to its protein misfolding, a ‘maladaptive’ pro-apoptotic ER stress has been reported [29]. In addition, mitochondrial dysfunction in AEC2 is a well-documented feature in IPF [18].

In this study, we analyzed the contribution of MAM proteins toward apoptosis and the ER–mito tethering in response to ER stress. Induction of Chop or pathological ER stress resulted in a decrease in ER–mitochondrial contacts in addition to a decrease in PACS2 as well as its interactor TRPV1, which in turn drove cells to apoptosis. Likewise, in alveolar epithelial cells stably expressing the IPF-causing mutation SPC^Δexon4^ as well as in AECII of IPF patients, a similar reduction in PACS2 as well as in TRPV1 protein levels was observed. Finally, we identified that treatment of ex vivo, three-dimensional lung slices or precision cut lung slices (PCLS) of IPF patients with the TRPV1-modulating drug capsaicin decreased apoptosis and restored both TRPV1 and PACS2 protein levels.

## Results

### Induction of Chop alone is sufficient to decrease ER–mitochondrial tethering and Pacs2 protein levels

**Fig. 1:**
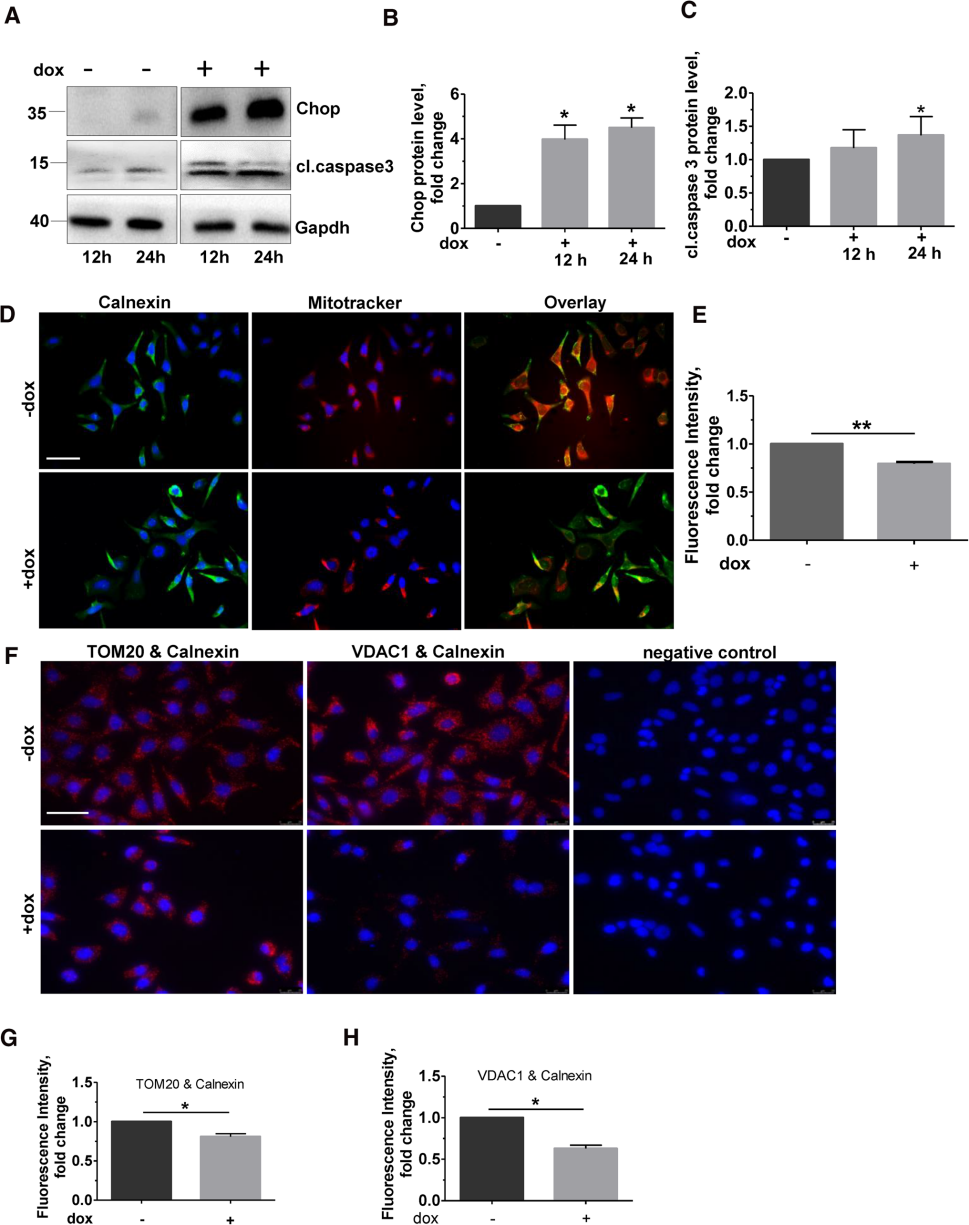
ER–mitochondria tethering is impaired upon Chop overexpression. **A** MLE12 cells were either not induced or were induced with 1 µg/mL doxycycline for Chop expression for 12 and 24 h, followed by immunoblots for the given proteins from total cell lysates. The positions of molecular weight markers are shown. **B** and **C** Quantification of significant increase in cl.caspase 3 protein levels (**C**) upon Chop induction (**B**) obtained from (**A**). Relative protein amounts were normalized to Gapdh and their level in −dox cells was set as one. **D** Representative immunoflourescence images of MLE12 cells either non-induced or induced with dox for 24 h for Chop expression. Staining for ER (calnexin, green) and mitochondria (MitoTracker red) is shown. Nuclei were stained with DAPI (blue), scale bar = 60 µm. **E** Fluorescence intensity of co-localization was quantified using ImageJ, and its intensity in −dox cells was set to one. **F** Proximity ligation assay with antibodies against calnexin and TOM20 or calnexin and VDAC1, followed by fluorescence microscopy images are shown; scale bar = 60 µm. **G** and **H** Fluorescence intensity was quantified using ImageJ, and its intensity in uninduced cells was set to one. Blots, stainings and analysis were performed from *n* = 3 and at least three technical replicates. Statistical significance is indicated as: *p ≤ 0.05, **p ≤ 0.01

In view of our previous work showing increased apoptosis of lung epithelial cells upon Chop induction, we asked if such Chop induction alone would result in altered ER–mitochondrial contacts. For this, as described before [17], we used mouse lung epithelial (MLE12) cells with stable, inducible (doxycycline, Dox +) overexpression of Chop (Fig. 1A, B), resulting in a significant increase in the terminal apoptosis marker cleaved caspase 3 (Fig. 1A, C), as well as cleaved Parp1 (Fig. S1A, B) in response to doxycycline treatment [17]. Co-immunofluorescence analysis for the ER protein calnexin and mitochondrial dye, MitoTracker, showed decreased co-localization of ER with mitochondria in dox-treated cells as compared to those with no dox (Fig. 1D, E). To study if the decreased co-localization between ER–mitochondrial markers could be attributed to a reduced ER–mitochondrial tethering, we performed proximity ligation assay (PLA) using antibodies against the ER protein calnexin and two mitochondrial proteins, namely the mitochondrial import receptor subunit translocase of outer membrane (TOM20) and the voltage-dependent anion-selective channel 1 (VDAC1). The fluorescence signal intensity significantly decreased in Chop-induced cells when a combination of ER–mitochondrial markers was used, clearly indicating that the proximity between ER–mitochondria indeed decreased upon Chop induction, as compared to no dox cells (Fig. 1F, G, H).

**Fig. 2:**
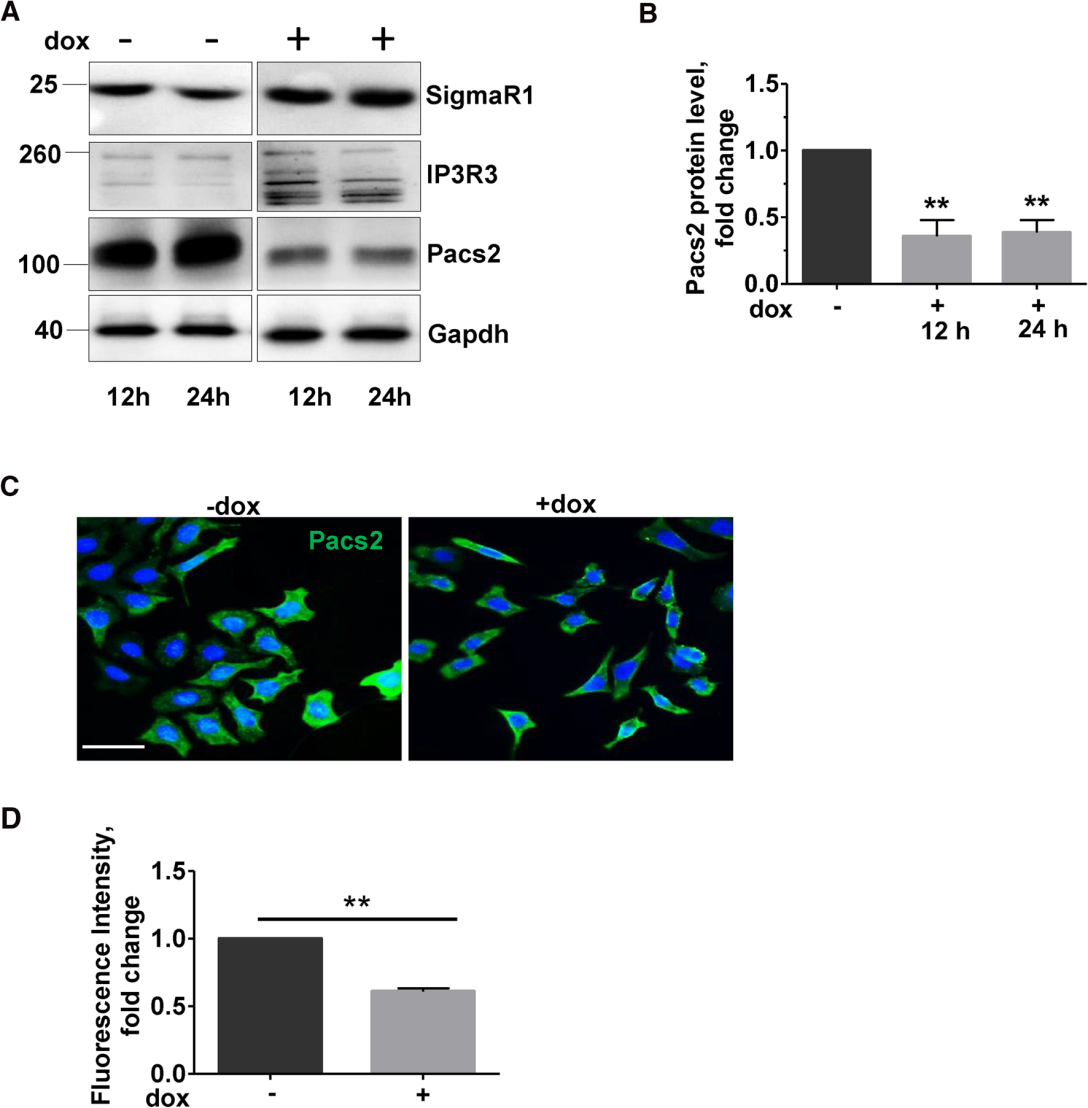
Pacs2 is decreased upon Chop overexpression. **A** MLE12 cells were either not induced or were induced with 1 µg/ml doxycycline for Chop expression for 12 and 24 h, followed by immunoblots for the given proteins from total lysates. **B** Quantification of significant decrease in Pacs2 protein levels upon Chop induction. Relative protein amounts were normalized to Gapdh and their level in −dox cells was set as one. **C** Representative immunoflourescence images of Pacs2 (green) in −dox or + dox-treated cells for 24 h. Nuclei were stained with DAPI (blue), scale bar = 60 µm. **D** Fluorescence intensity was quantified using ImageJ, and its intensity in −dox cells was set to one. Blots and analysis were performed from *n* = 3 experiments and statistical significance is indicated as: **p ≤ 0.01

We next asked if the decreased ER–mitochondrial tethering is due to an alteration in MAM proteins. For this, we performed immunoblots for SigmaR1, IP3R3 and Pacs2. Of the MAM proteins analyzed, we observed a significant decrease in Pacs2 protein (Fig. 2A, B) upon 12 and 24 h of Chop induction. Supporting this, immunofluorescence analysis also revealed a significant decrease in Pacs2 protein in Chop-induced cells (Fig. 2 C, D). Further, to rule out any effects of doxycycline treatment on ER–mitochondrial tethering, we also treated healthy MLE12 cells with doxycycline and analyzed for Pacs2 protein levels. As indicated in Fig. S2 A, doxycycline treatment itself did not affect the protein levels of Pacs2 or Chop nor did it affect ER–mitochondrial tethering (Fig. S2B). The specificity of Pacs2 antibody was confirmed via siRNA mediated knockdown experiments (Fig. S3A, B).

### Pacs2 is required to maintain ER–mitochondrial tethering in cells with Chop induction

**Fig. 3:**
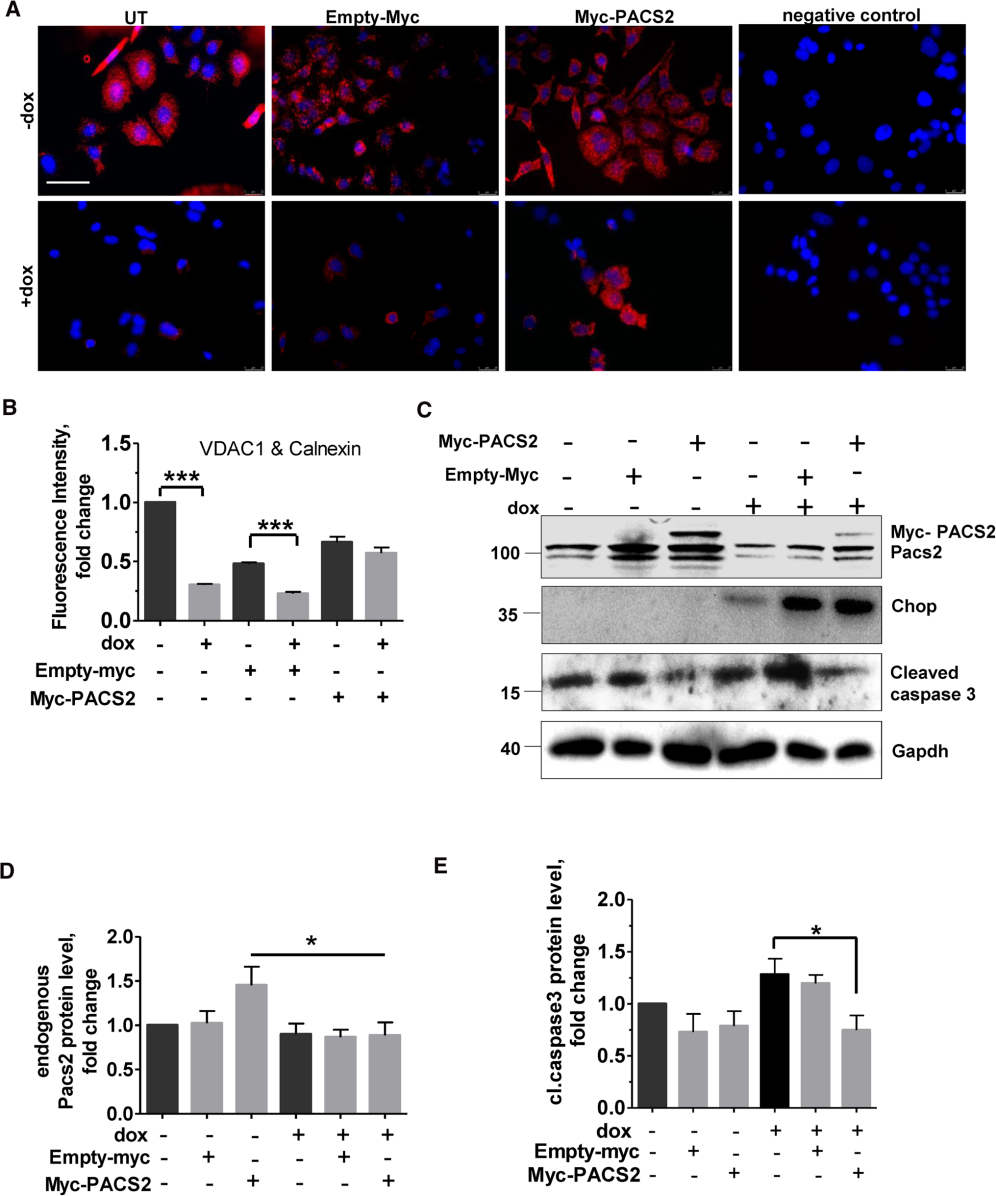
Pacs2 overexpression rescues phenotype of Chop-induced cells. MLE12 cells were either mock transfected, or transfected with empty-Myc or Myc-PACS2 for 4 h, followed by no dox (− dox) or with dox (+ dox, 12 h) treatment for Chop overexpression, followed by (**A**) proximity ligation assay with antibodies against calnexin and VDAC1 and subsequent fluorescence microscopy, scale bar = 60 µm. **B** Fluorescence intensity was quantified using ImageJ, its intensity in untreated −dox cells was set to one. **C** Immunoblots for the indicated proteins. **D** Relative protein levels of Pacs2 and **E** cleaved caspase 3 were normalized to Gapdh and their level in −dox cells was set as one. Representative images and stainings from *n* of 3 independent experiments are shown, statistical significance is indicated as: *p ≤ 0.05, ***p ≤ 0.001

Since Pacs2 protein controls ER–mitochondria communication, we hypothesized that the decreased ER–mitochondrial tethering that was observed in Chop-induced cells is a result of decreased Pacs2 protein levels. Hence, to test this, we over-expressed myc-tagged Pacs2 in our Chop engineered MLE12 cells, followed by doxycycline treatment and Chop induction. In accordance with our hypothesis, Pacs2 overexpression helped to maintain ER–mito tethering in doxycycline-treated, Chop-induced cells, as compared to Chop-induced cells without Pacs2 overexpression (Fig. 3A, B). Interestingly, in the same cells, PACS2 overexpression in Chop-induced cells also helped to reduce the extent of apoptosis, as displayed by a decrease in the terminal apoptosis marker, cleaved caspase 3 (Fig. 3C, D, E).

### Modulating Pacs2–TRPV1 axis rescues Chop-induced cells from apoptosis and ER–mitochondrial tethering

**Fig. 4:**
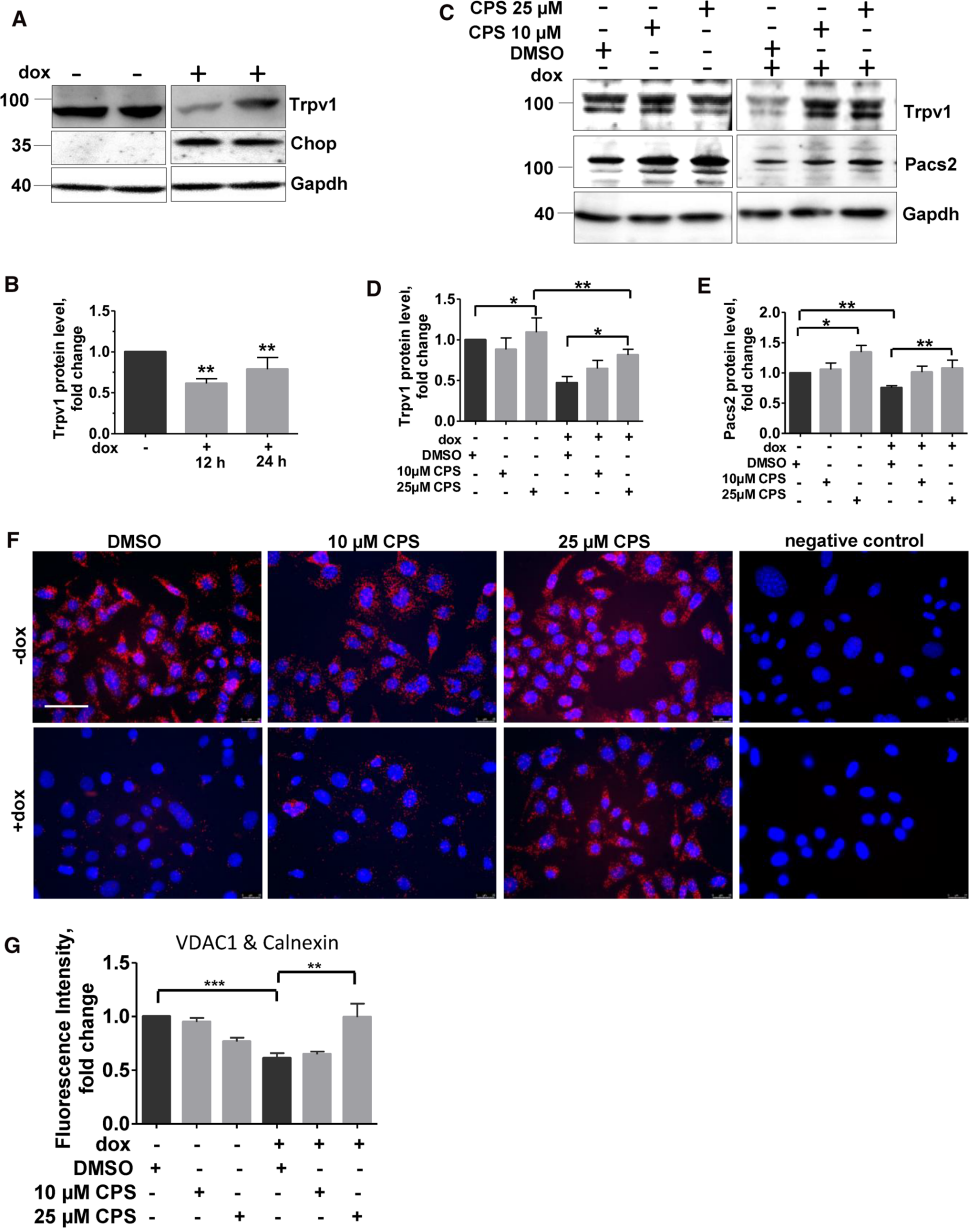
Modulation of Trpv1 improves ER–mitochondrial tethering in cells overexpressing Chop. **A** Immunoblot analysis of Trpv1 in total cell lysates of MLE12 cells untreated or treated with dox for Chop expression for 12 and 24 h. **B** Relative protein level of Trpv1 were normalized to Gapdh and its level in −dox cells was set as one. **C** MLE12 cells were either left untreated or treated with dox for 12 h, followed by treatments with DMSO or CPS at indicated concentrations followed by immunoblots for the indicated proteins. **D** and **E** Quantification of Trpv1 (**D**) and Pacs2 (**E**) protein expression is shown. Relative protein amounts were normalized to Gapdh and their mean value in respective DMSO-treated controls was set as one. **F** Representative fluorescence images following proximity ligation assay with antibodies against calnexin and VDAC1, and cells treated with CPS at the indicated concentrations or DMSO upon Chop induction are shown, scale bar = 60 µm. **G** Fluorescence intensity was quantified using ImageJ, its intensity in −dox, DMSO-treated cells was set to one. ‘*n*’ of three independent experiments were performed and statistical significance is indicated as: *p ≤ 0.05, ***p ≤ 0.01, ***p ≤ 0.001

Chemical modulators of Pacs2 function are currently not available. We hence asked for mechanisms that may interfere with Pacs2 functions. A careful literature search revealed that Pacs2 is ubiquitinated and degraded by cellular inhibitor of apoptosis (cIAP1/2) and their cellular levels inversely correlate with apoptosis [11]. We hence analyzed for protein levels of cIAP1/2 in Chop-induced cells. However, we did not find a notable regulation of these proteins (Fig. S4A, B), indicating that in response to Chop, Pacs2 is not influenced by these proteins. Next, it has been reported that Pacs2 is an interacting partner of the transient receptor potential cation channel subfamily V member 1 (TrpV1/ TRPV1) [30], a ligand gated ion channel, the activation of which causes Ca^2+^ and Na^+^ influx, with a higher selectivity for Ca^2+^ over Na^+^ [14]. Supporting this, via immunoprecipitation analysis, we also observed an interaction between Pacs2 (Myc-Pacs2) and Trpv1 in MLE 12 cells (Fig. S5). We next asked if Trpv1 is altered upon Chop induction. Indeed, immunoblotting analysis revealed that Trpv1 is decreased in cells overexpressing Chop (Fig. 4A, B). This made us hypothesize that, since Trpv1 and Pacs2 are interacting partners, modulation of Trpv1 may influence Pacs2 protein levels as well.

Trpv1 is activated and its protein levels may increase in response to vanilloids like capsaicin (CPS), a natural alkaloid and analgesic found in chili peppers. We treated alveolar epithelial cells overexpressing Chop with 5, 10 and 25 µM CPS for 8 h. Non-toxic concentrations as determined by LDH assay (Fig. S6) were further considered. In cells with no dox treatment, CPS treatment did result in a slight and significant increase in Trpv1 only at a dose of 25 µM as compared to DMSO treatment. In dox + cells with Chop induction, a consistent and significant increase in Trpv1 protein levels was observed in response to CPS (Fig. 4C, D). Interestingly, CPS treatment also resulted in an increase in Pacs2 protein levels in dox and non-dox treated cells (Fig. 4C, E). Further, in situ PLA also revealed an increased ER–mito tethering upon treatment with 25 µM CPS in Chop overexpressing cells (Fig. 4F, G).

**Fig. 5:**
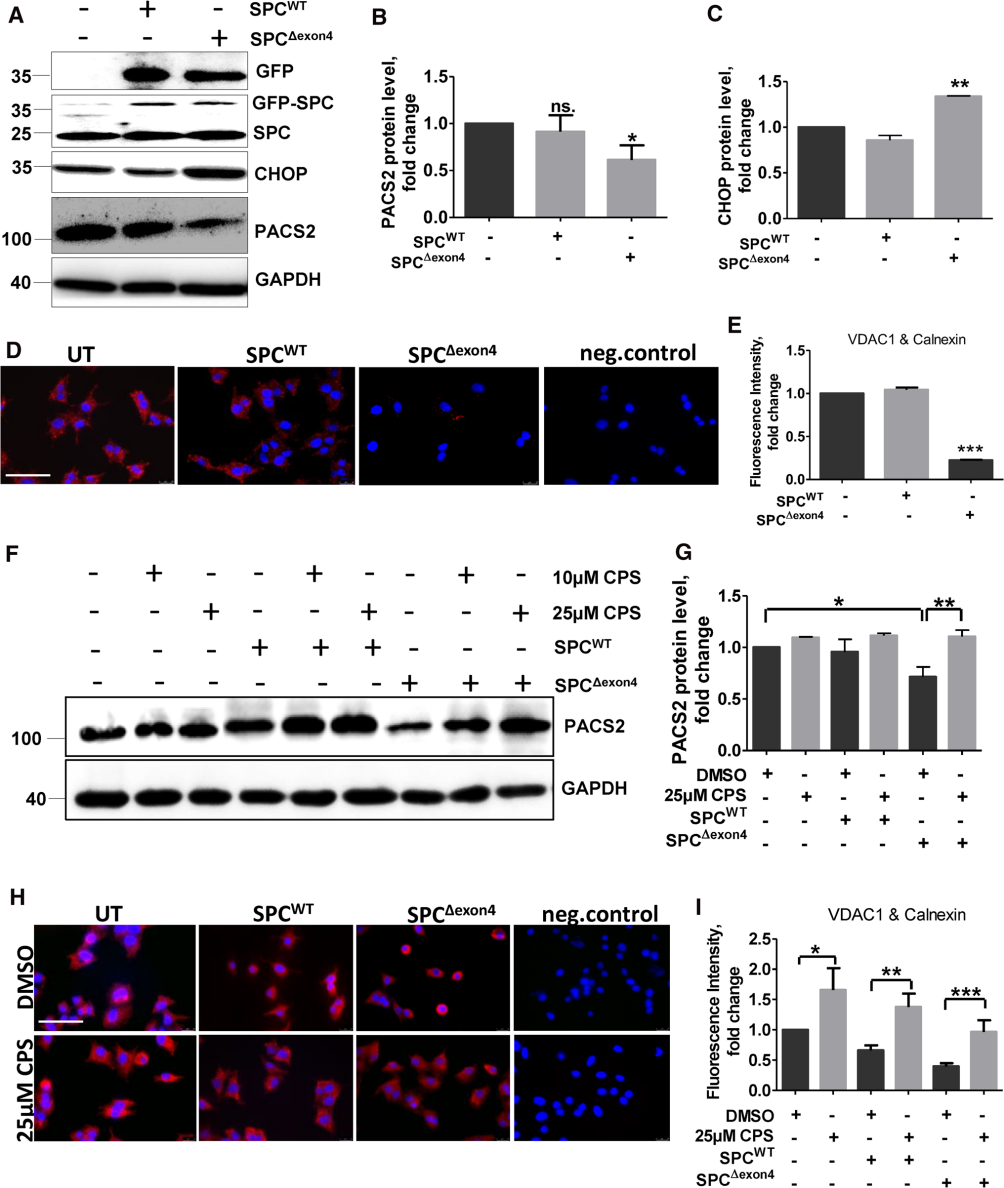
Modulating PACS2–TRPV1 axis with CPS rescues ER–mitochondrial tethering and PACS2 protein in cells overexpressing SPC^Δexon4^. **A** MEL188 cells were stably transfected with either SPC^WT^ or SPCΔ^exon4^ plasmids or left un-transfected, followed by immunoblots for the given proteins. **B** and **C** Quantification of PACS2 (**B**) and CHOP (**C**) protein levels are shown. Relative protein amounts were normalized to GAPDH and their mean value in untransfected cells was set as one. **D** Representative fluorescence microscopy images following proximity ligation assay with antibodies against calnexin and VDAC1 in SPC^WT^ or SPCΔ^exon4^ overexpressing cells, scale bar = 60 µm. **E** Fluorescence intensity was quantified using ImageJ, and its intensity in untransfected cells was set to one. **F** Cells stably expressing SPC^WT^or SPCΔ^exon4^ or control cells were treated with DMSO or CPS, followed by immunoblotting of PACS2 and GAPDH. **G** Quantification of PACS2 protein expression is shown. Relative protein amounts were normalized to GAPDH and its mean value in control cells was set as one. **H** Fluorescence microscopy images following proximity ligation assay with antibodies against calnexin and VDAC1 in control cells or in cells overexpressing SPC^WT^ or SPCΔ^exon4^, followed by DMSO or CPS treatments at the indicated doses, scale bar = 60 µm. **I** Fluorescence intensity was quantified using ImageJ, and its intensity in untreated control cells was set as one. ‘*n*’ of three independent experiments were performed and statistical significance is indicated as: *p ≤ 0.05, **p ≤ 0.01, ***p ≤ 0.001

ER stress has been previously reported in cells and in mice overexpressing SP-C^Δexon4^. In humans, a heterozygous mutation of A to G in the first base of intron 4 of the human SP-C gene (c.460 + 1A > G) results in the absence of mature SP-C protein [41]. The underlying reason is a spliced deletion of exon4 resulting in the removal of the conserved cysteine in the C-terminal flanking propeptide, which leads to the generation of a misfolded SP-C protein, with toxic gain of function. Patients with such mutation are reported to develop familial interstitial lung disease [28]. In vitro, overexpression of SPC^Δexon4^ resulted in increase in markers of unfolded protein response (UPR) [27]. Hence, we next aimed to overexpress SPC^Δexon4^ in vitro and analyze ER–mitochondrial tethering under these conditions, to understand if ER–mito tethering is also affected in a condition with a broader ER-stress response as observed in SPC^Δexon4^ driven disease. For this, we first performed gene splicing by overlap extension (gene SOEing) PCR to generate a plasmid encoding SPC^Δexon4^ (Fig. S7A, B) as described in methods. Either the SPC^WT^ or the SPC^Δexon4^ plasmid was stably transfected in human melanoma cell lines (MEL188). We chose MEL 188 cell line, as these cells have human origin, possess lamellar bodies and express the 21 kDa pro SP-C protein. Like MLE 12 cells, these cells also do not cleave SP-C to its mature form. Interestingly, as compared to control cells or SPC^WT^ overexpressing cells, SP-C^Δexon4^ overexpression resulted in an increase in Chop protein indicative of ER stress (Fig. 5A, C). Further, a decrease in PACS2 protein was also observed (Fig. 5 A, B) in addition to a decreased ER–mitochondrial tethering (Fig. 5D, E). Like in Chop overexpressing alveolar epithelial cells, treatment of SPC^Δexon4^ cells with CPS also resulted in an increase in PACS2 protein levels (Fig. 5F, G). On the same line, ER–mito tethering increased in response to 25 µM CPS as compared to DMSO treatment in these cells (Fig. 5H, I). Cells overexpressing SP-C^WT^ did not show any differences in PACS2 protein levels, nor did they exhibit any decrease in ER–mitochondrial tethering. Results until this point indicated that singular Chop overexpression, as well as a pathological ER stress reaction driven by SPC^Δexon4^, resulted in decreased protein levels of PACS2 and a decrease in ER–mito tethering. The TRPV1-modulating drug CPS was fully capable of reversing these detrimental effects.

**Fig. 6:**
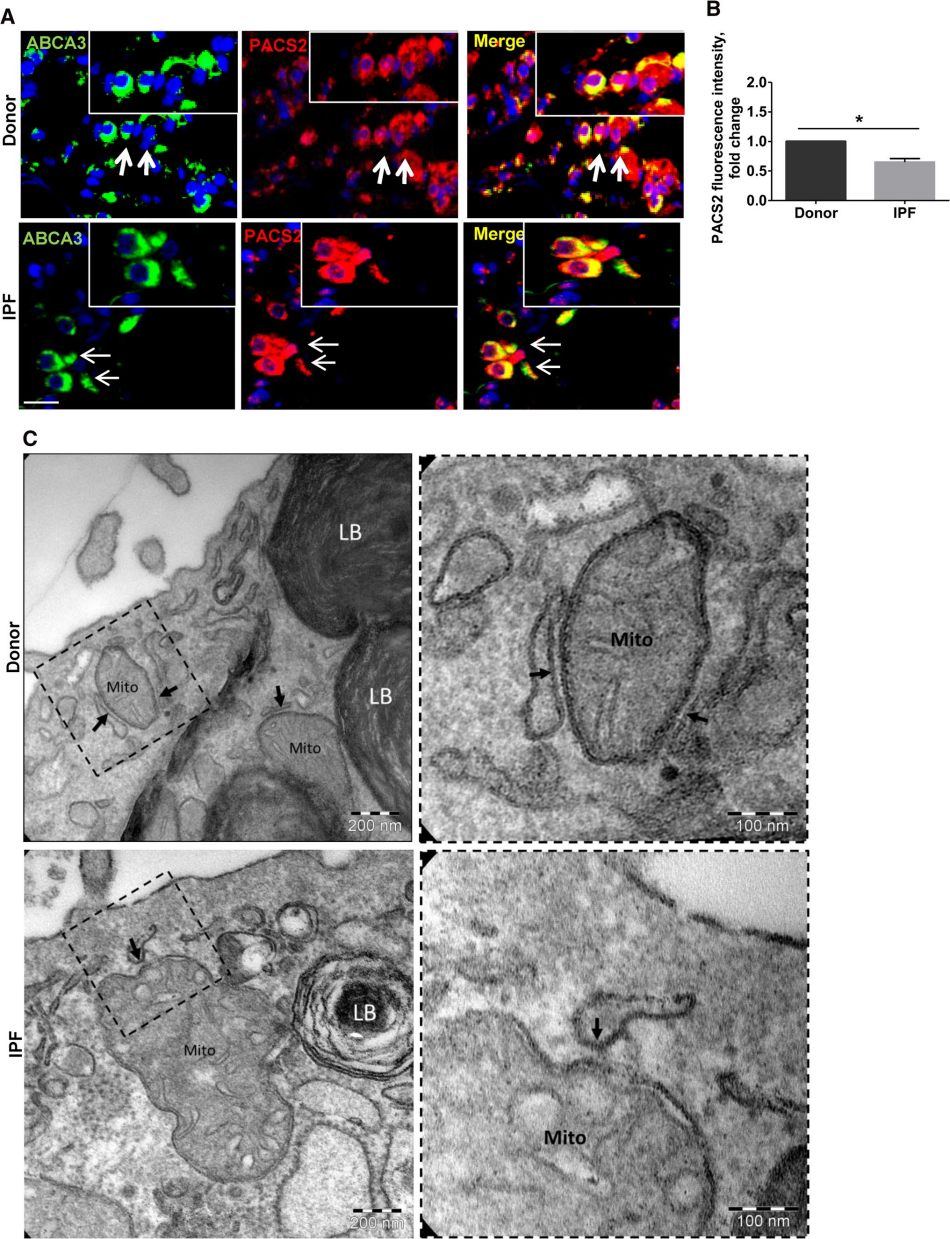
PACS2 protein and ER–mitochondrial tethering are decreased in IPF AECII. **A** Representative immunofluorescence images for PACS2 (red) and ABCA3 (green) in IPF and Donor lung sections, and nuclei were stained with DAPI (blue) scale bar = 25 µm. **B** Fluorescence intensity of PACS2 was quantified using ImageJ, and its intensity in Donor sections was set as one. Lung sections from seven IPF to seven Donors were used for stainings. Statistical significance is indicated as: *p ≤ 0.05. **C** Representative transmission electron microscopic images from alveolar epithelial type II cells (characterized by the presence of lamellar bodies (LB)) are shown. At the ultrastructural level, the appearance of the contact zones between ER and mitochondria (Mito) in IPF differed from the healthy control. The closest contacts (arrows) appeared to be rather point shaped with the ER piston-like widened in IPF. In healthy controls, the contacts were in general elongated and more laminar so that the ER and mitochondrial membranes had a parallel run

Further, since ER stress is a well-documented pathomechanistic feature of IPF AECII [18], we sought to analyze the MAM proteins in IPF patient lungs as compared to healthy donor (Donor) lungs. Immunofluorescence analysis revealed a significant decrease in PACS2 in the AECII (ABCA3-positive cells) of IPF lungs (Fig. 6A, B). Next, we also analyzed the ultrastructure of the contacts between ER and mitochondria in AECII of IPF and Donor lungs. In some areas, the membrane of the ER approached the outer membrane of the mitochondria in a way that there were no ribosomes between them and the membranes were separated from each other by a very thin leaflet of cytosol. In these contact regions, the distance between the membranes was only a few nanometers. Based on these considerations the MAMs could be easily identified by ultrastructural criteria. As indicated in Fig. 6C, electron microscopy analysis demonstrated that in IPF AECIIs, the contact zones were more point shaped and the ER appeared to be locally widened. In AECII from healthy-appearing lung tissue (Donors), the contact zones between ER and mitochondria were rather laminar than point shaped and the ER was not widened.

**Fig. 7:**
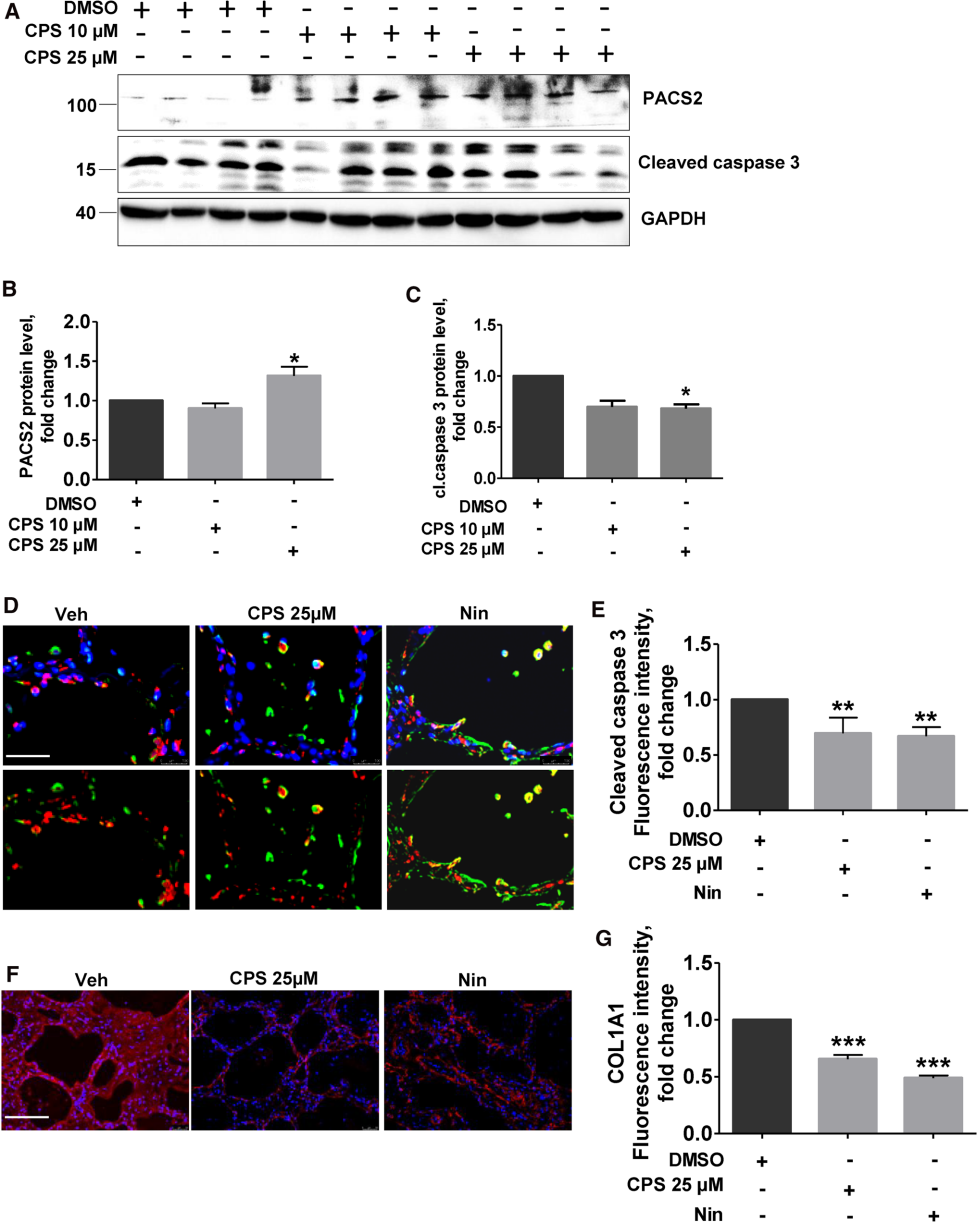
Modulation of TRPV1 decreases apoptosis and COLA1A level in PCLS of IPF patients. **A** PCLS from explanted IPF patient lungs were treated with either DMSO or CPS with the indicated concentrations for 8 h, followed by immunoblot analysis for PACS2, cleaved caspase 3 and GAPDH. **B**-**D** Quantification of PACS2 (**B**) and cl.caspase3 (**C**) protein levels is shown. Relative protein amounts were normalized to GAPDH and their mean value in DMSO-treated PCLS was set as one. **D** Immunofluorescence staining for cl.caspase 3 (red) in AECII (ABCA3, green) on IPF PCLS upon Veh, CPS or Nin treatment for 8 h. Nuclei were stained with DAPI (blue), scale bar = 60 µm. **E** Fluorescence intensity of cleaved caspase 3 in ABCA3-positive cells was quantified using ImageJ, and its intensity in DMSO-treated cells was set as one. **F** Immunofluorescence staining for COLA1A on IPF PCLS upon Veh, CPS or Nin treatment for 8 h. Nuclei were stained with DAPI (blue), scale bar = 60 µm. **H** Fluorescence intensity of COLA1A was quantified using ImageJ, and its intensity in DMSO-treated cells was set as one. Stainings and analysis were performed in PCLS performed from three IPF patients and significance is indicated as: *p ≤ 0.05, **p ≤ 0.01, ***p ≤ 0.001

To understand the effects of PACS2–TRPV1 axis modulation in IPF, we treated precision cut lung slices (PCLS) of IPF patients with CPS. A significant increase in PACS2 protein levels was observed in IPF PCLS treated with 25 µM CPS as compared to DMSO-treated PCLS (Fig. 7A, B). In addition, a reduction in the apoptosis marker cleaved caspase 3 (Fig. 7A, C) was also observed. In full support, quantification of immunofluorescence stainings for cleaved caspase 3 also showed a decrease in cleaved caspase 3 stainings in CPS-treated PCLS (Fig. 7D, E).

Further, we asked if treatment of IPF PCLS with CPS would have any effect on extracellular matrix protein, collagen1A1 (COL1A1), which is usually higher in IPF tissues due to the excessive deposition of extracellular matrix. Immunofluorescence analysis revealed a significant decrease in COL1A1 in IPF PCLS treated with 25 µM CPS as compared to DMSO-treated PCLS (Fig. 7F, G).

## Discussion

**Fig. 8:**
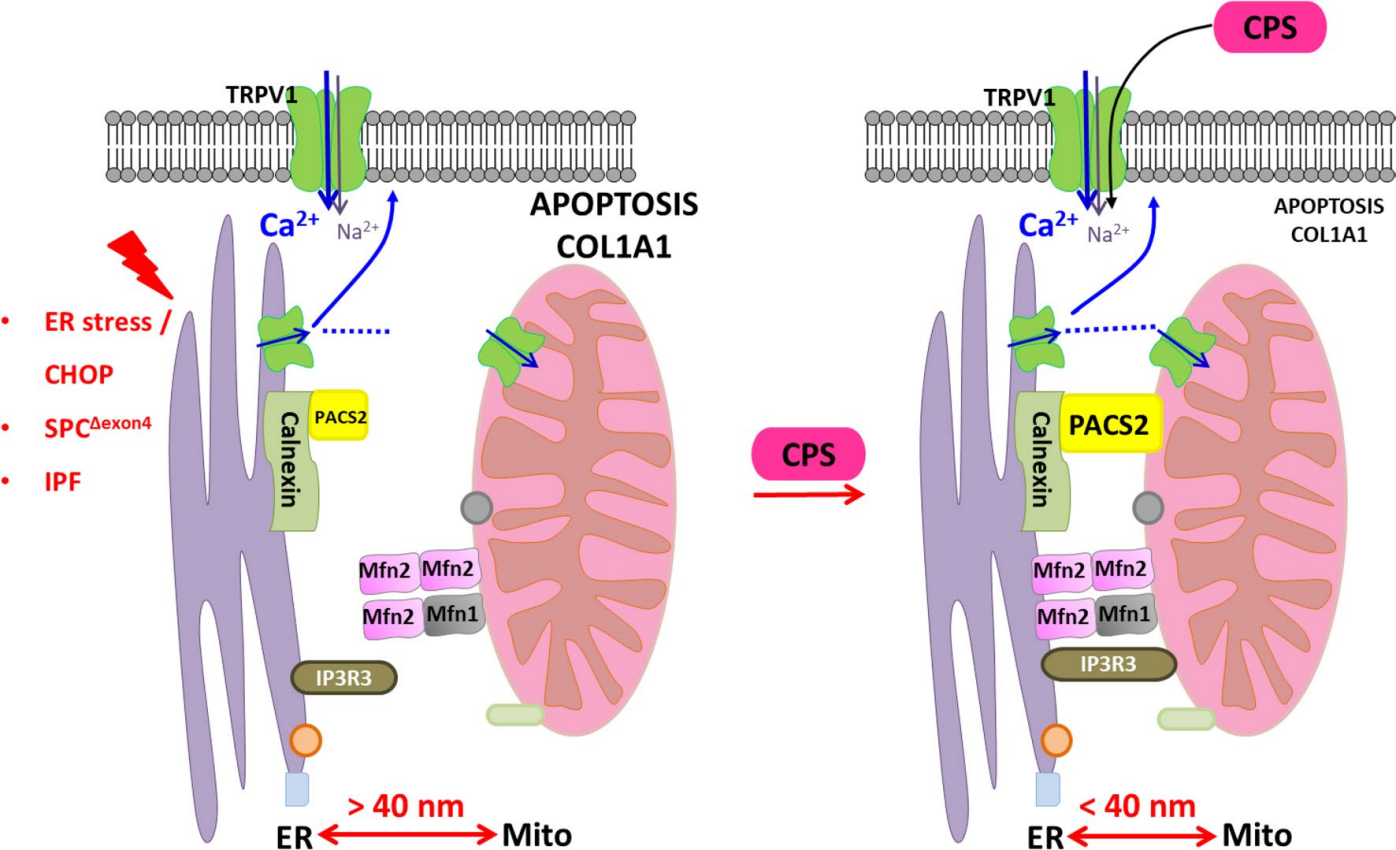
PACS2–TRPV1 axis is required for ER–mitochondrial tethering during ER stress and lung fibrosis. Cartoon summarizing the results of this study: left side: upon ER stress/CHOP induction, SPC^Δ^^exon4^ overexpression or in IPF AECII where persistent pro-apoptotic ER stress is observed, PACS2 protein is severely decreased and, therefore, a reduction in ER–mitochondrial tethering is observed alongside with severe AECII apoptosis. Upon treatment with CPS, a TRPV1 modulator, PACS2 protein levels are restored, ER–mitochondrial tethering is rescued together with a decrease in AECII apoptosis and COL1A1

In this study, we show that ER–mito tethering is disrupted in three different conditions: 1. upon induction of the terminal ER stress associated apoptosis marker Chop in murine alveolar epithelial cell lines, 2. upon overexpression of the disease-causing SPC^Δexon4^ mutation in human melanoma cells and 3. in the lungs and PCLS of patients with IPF. In addition, we were able to also show that downregulation of Pacs2 protein is largely responsible for the disruption of ER–mitochondrial tethering, which was rescued by overexpressing Pacs2 in Chop-induced cells (Fig. 8). Additionally, we identified that Pacs2 interacts with Trpv1 and modulation of Trpv1 by CPS rescued ER–mitochondrial tethering in addition to Pacs2 protein levels. Supporting this, in IPF AECII, we observed few point-like contacts rather than extended sheet-like contacts, as observed in healthy lung regions. Treatment of PCLS of IPF patients with CPS likewise resulted in decreased apoptosis of AECII and deposition of collagen 1, highlighting the significance of the PACS2–TRPV1 axis in IPF.

Chronic ER stress and mitochondrial dysfunction in AECII are pivotal pathomechanistic features driving apoptosis in sporadic as well as familial forms of IPF. CHOP is increased particularly in the AECII of sporadic IPF patient lungs alongside with a remarkable increase in other major ER stress signature molecules, GRP78, ATF4, ATF6α and spliced XBP1 [18, 20]. It has long been indicated that CHOP is a crucial regulator of ER stress-induced apoptosis [43]. Further, we and others showed that Chop overexpression results in AECII apoptosis [17], while its knockdown protects cells from ER stress-induced apoptosis [31]. Likewise, overexpression of SFPTC gene BRICHOS domain mutations (familial form of IPF) in epithelial cell lines in vitro resulted in the upregulation of several genes of the unfolded protein response pathway (UPR) [24]. Further, mice overexpressing Sftpc^C121G^ mimicking clinical cases of SPC BRICHOS mutations were recently shown to elaborate ER stress markers including Chop [15]. In full support, we here showed that MEL188 cells overexpressing BRICHOS domain mutation (SPC^Δexon4^) display significantly upregulated CHOP protein. Further, in AECII of IPF patients, where severe and pro-apoptotic ER stress has been well documented [18], the ER–mito tethering, as shown by PLA and EM in this study, was found to be greatly reduced, again emphasizing the role of ER stress on ER–mitochondrial contacts in this cell type.

One intriguing observation of our study is altered PACS2 protein in these ER stress models and in IPF lungs. PACS2 is a multifaceted sorting protein that transfers several cargo proteins to respective organelles and is therefore an important protein that is involved in membrane trafficking as well as in maintaining ER–mitochondrial communication [39]. It was shown to bind to and transport Bid protein from the cytoplasm to mitochondria, further activating cell death pathways [21]. A direct regulation between CHOP and PACS2 proteins has not been suggested so far. It was, however, reported that, in response to ER stress inducers like staurosporine and tunicamycin, PACS2 protein is translocated from cytosolic and ER-enriched light membrane fractions to mitochondria containing heavy membrane fractions [37]. Of note, PACS2 protein is a crucial protein and a key regulator of MAMs. Depletion of PACS2 was shown to also promote apoptosis and this was linked to a defect in the mitophagy pathway [26]. From our study, we can infer that downregulation of PACS2 is at least in part responsible for the decreased apposition of ER and mitochondria as well as for the increased apoptosis in our models.

Both PACS1 and PACS2 were shown to be involved in the trafficking of polycystin-2, a member of the TRPP2 ion channel family [19]. On the other hand, both wild-type and mutant PACS2 (pGlu209Lys) interact with TRPV1 at different levels, implicating the importance of PACS2 in channelopathies [30]. Such PACS2–TRPV1 interaction is also observed in our current study in healthy MLE12 cells and a decrease in Trpv1 protein levels was observed in cells overexpressing Chop. Pharmacological inhibitors/activators for PACS2 are not available till date. But stabilizing PACS2–TRPV1 axis greatly influenced the cellular outcome in our models. Both Chop and SPC^Δexon4^ overexpressing cells showed improved ER–mito tethering and restoration of PACS2 protein levels upon exposure to the TRPV1-modulating drug CPS.

CPS is an alkaloid found in chili peppers and is the cause of burning sensation when in contact, but has a wide range of medical benefits. Its topical application is especially appreciated for pain therapies arising from metabolic diseases like diabetes [38] and high concentration capsaicin patches are approved and well tolerated for neuropathic pain [1]. Its anti-hypertensive [13], anti-tumorigenic [25] and anti-inflammatory [9] properties are well documented. It has been reported to show inhibitory effects in hepatic fibrosis [36] and to prevent renal damage in acute kidney injury [42]. In the lung, CPS has been shown to reduce pulmonary remodeling and collagen fibrils in vessels and lung tissues of animals with chronic lung inflammation [32]. Further, one study showed that low doses of CPS alleviated bleomycin-induced lung fibrosis via inhibiting ERK1/2/eIF3a signaling in the alveolar epithelial cells [22]. Supporting this, our study now shows that treatment of IPF PCLS with CPS rescued cellular protein levels of Pacs2, reduced cleaved caspase 3 levels indicating reduced apoptosis in alveolar epithelial cells and also reduced collagen deposition in human IPF PCLS. CPS in fact targets TRPV1 channel which is also known as CPS receptor. CPS activates TRPV1 making it more permeable to cations, leading to analgesic effect mostly due to channel desensitization [8]. TRPV1 is increased in bleomycin-induced lung fibrosis in guinea pigs [12] and TRP channels in general were suggested to contribute to lung repair processes [7]. However, TRPV1 modulation in experimental lung fibrosis had not been studied before, but our results indicate a beneficial role of CPS via modulation of TRPV1 and restoration of PACS2 protein levels and ER–mito tethering. In conclusion, our study shows for the first time that disturbed ER–mito tethering, induced by ER stress in vitro and ex vivo, can be overcome by application of CPS and results in increased Pacs2 protein levels, increased ER–mito tethering, reduced alveolar epithelial apoptosis and reduced collagen expression. Our study poses two possibilities: 1. CPS may increase TRPV1 protein levels or its channel activity or 2. CPS may inhibit the degradation of TRPV1, thereby stabilizing the protein, as has been suggested before [8]. In-depth studies are required to answer which of these possibilities exist upon CPS treatment in lung fibrosis. Further, our study does not allow a definite statement with regard to the clinical effects and the therapeutic efficacy of a modification of the TRPV1–PACS2 axis in IPF patients, but our results obtained by use of CPS in human IPF PCLS studied ex vivo certainly provide important argument for further development of drugs improving ER–mitochondrial communication.

## Materials and methods

Lung tissue samples were obtained from patients with sporadic IPF and non-diseased control subjects. Explanted lungs or lobes were obtained from the Department of Thoracic Surgery, Vienna, Austria, and were collected in the frame of the European IPF Registry/Biobank (eurIPFreg/bank). Human lung samples were provided by the Universities of Giessen and Marburg Lung Center (UGMLC) Biobank, the member of the German Centre for Lung Research (DZL) Platform Biobanking. All IPF diagnoses were made according to the American Thoracic Society (ATS)/European Respiratory Society (ERS) consensus criteria. The study protocol was approved by the Ethics Committee of the Justus-Liebig University Giessen (111/08 and 58/15). Formalin-fixed lung tissue blocks were obtained from eight patients with IPF (mean age 55 ± SD: 10.28) and eight non-diseased control subjects (Donors; mean age 39 ± SD: 13.91).

### Cell culture

Mouse lung epithelial cell line (MLE-12) was obtained from ATCC, Manassas, USA. Human melanoma cell lines (MEL188) were a kind gift from Prof. Dr. Timothy E. Weaver, Cincinnati Children’s Hospital, Ohio, USA. Inducible mouse lung epithelial cells for CHOP (MLE-12/pBI-L-CHOP) were grown and cultured as described before [17]. 24 h after thawing, fresh medium with selection antibiotics (1 µg/mL doxycycline, 100 µg/mL hygromycin, 100 µg/mL geneticin) was added to induce CHOP expression for 6, 12 or 24 h (+ dox). Cells grown in normal growth medium (without antibiotics or doxycycline) for similar time points were treated as control group (− dox). All cells were grown in tissue culture plates in full medium based on Dulbecco’s modified Eagle medium/Nutrient Mixture F-12 (DMEM-F12 Medium). Transient transfection was performed in a six-well dish using 4 µg PACS2-GFP (OriGene, Rockville, USA) or empty-GFP using 4 µl Lipofectamine^®^ 2000 Reagent (Invitrogen, Germany) per well or with non-targeting (NT) or Pacs2 siRNA (both from Santa Cruz) for 48 h as per manufacturer’s instructions.

### Immunoblotting and immunofluorescence

Cells were harvested, sonicated and processed according to previously described protocols. PCLS were homogenized in lysis buffer with protease inhibitor (PMSF) using Precellys^®^ 24 homogenizer (Bertin Technologies, Germany) as described before for lung tissues [16] and using standard protocols for immunoblots. ECL Enhanced Chemiluminescence (ECL) Chemostar imager (Intas, Germany) was used to visualize bands, and relative expression level of proteins in blots was calculated by measuring density of bands using Image J software (version 1.52a, NIH, USA). Integrated density values of the respective proteins were normalized against those of ß-actin or GAPDH values. The values of controls were then assumed as 1 and the fold change of a target protein was calculated. All antibodies used for immunoblotting are outlined in Table 1. Immunofluorescence was performed on cells or on 3 µM formalin-fixed, paraffin-embedded PCLS that were deparaffinized and immunostainings were performed using antibodies given in Table 1, following protocols as described before [16]. Microscopy was performed using Leica M205 FA fluorescence stereoscope (Leica Microsystems, USA) and LAS-X-Core Software (3.7.4. version, LAS X Life Science, USA). Immunofluorescence images were quantified using Leica LAS X LS software on dive to ten randomly selected regions per mouse or human sections and represented as mean fluorescence intensity.

**Table 1:** Antibodies used in this study

Name	Catalog no	Company
Immunoblotting		
ß-actin	ab8227	Abcam
CHOP	5554 s	Cell signaling
Cleaved caspase 3	9662 s	Cell signaling
Cleaved PARP1	ab32064	Abcam
GAPDH	2118 s	Cell signaling
GFP	ab5450	Abcam
IP3R3	NBP 1-21,400	Novus
LC3B	ab48394	Abcam
p62/SQMSTM 1	P0067	Sigma
PACS2	19,508-1-AP	Proteintech
SigmaR1	15,168-1-AP	Proteintech
TRPV1	PA1-29,421	Invitrogen
Pro SP-C	AB3786	MerckMillipore
Immunofluorescence		
ABCA3	WAMB-ABCA3-17	Seven hills
Calnexin	sc6465	Santa Cruz
Cleaved caspase 3	9662 s	Cell signaling
COL1A1	600-401-103-0.5	Rockland
PACS2	19,508-1-AP	Proteintech
VDAC1	10,866-1-AP	Proteintech
Tom20	sc11415	Santa Cruz

### Electron microscopy

The lung samples investigated by electron microscopy were taken from prior studies [4, 23, 34]. In brief, the samples were fixed by immersion using a mixture of 1.5% glutaraldehyde and 1.5% paraformaldehyde in 0.15 M HEPES buffer. To increase the contrast of the membranes, the tissue was stained en bloc based on the rOTO (reduced osmium tetroxide–thiocarbohydrazide–osmium tetroxide) protocol as described previously [3, 6]. Ultrathin sections were cut at a thickness of approximately 60 nm and investigated using a transmission electron microscope (Morgagni, Philips, Eindhoven, The Netherlands).

### Precision cut lung slices (PCLS)

1.5% low melting agarose (maintained at 37 °C) was filled in each segment of explanted human IPF lung and was allowed to cool on ice for 30 min for the agarose to solidify. Vibrating blade microtome (Thermo Fisher) was used to section blocks of tissue filled with agarose. About 500 μm thick sections were made and were cultured in RPMI medium without phenol red supplemented with 2% FCS, 1% penicillin/streptomycin and 1% L-glutamine. PCLS were left for 24–48 h in a cell culture incubator. Before treatments, PCLS were washed with PBS and drug treatments were performed as described below.

### Drug treatments

CHOP-overexpressing MLE12 cells (± dox for 12 h) or MEL188 cells stably overexpressing SPC^WT^ or SPC^Δexon4^ were treated for 8 h with capsaicin (5 µM,10 µM, 25 µM, Sigma, #M2028) or vehicle (DMSO) before processing them for immunoblotting or PLA. PCLS were treated for 24 h with capsaicin (10 µM, 25 µM) or nintedanib (2 μM, Sigma, #SML2848). Sterile filtered DMSO served as vehicle control. About four to five IPF patient PCLS were cultured in one well of a six-well dish for each treatment group and ‘*n*’ of three IPF patient PCLS were used.

### Cloning

RNA was extracted from Donor lungs using Trizol. 2 µg of RNA sample was reverse-transcribed to cDNA using Omniscript Reverse Transcription Kit (Qiagen) and Oligo-dT primers (Applied Biosystem). 2 µg cDNA was then amplified using high-fidelity DNA polymerase and SPC^WT^ primers (Metabion) as outlined in online supplement. Generation of MEL188 cells stably expressing SPC^WT^ or SPC^Δexon4^ is described in the online supplement.

### Proximity ligation assay

Proximity ligation assay (PLA) was performed for in situ detection of the distance between ER and mitochondria (distance < 40 nm) using Duolink^®^ In Situ Orange Starter Kit Goat/Rabbit (Sigma Aldrich, Germany) following the manufacturer’s instructions. Briefly, two primary antibodies raised in different species (goat anti-calnexin as an ER marker and rabbit anti-TOM20 or rabbit anti-VDAC1 as mitochondrial markers, antibody information in Table 1) were used. A pair of oligonucleotide-labeled secondary antibodies (PLA probes) was added to bind to the primary antibodies. Hybridizing connector oligos were then used to join the PLA probes. Upon close proximity, a circular DNA template is formed by ligase activity resulting in rolling-circle amplification. Amplified signal tethered to the PLA probe was generated that allowed signal detection. The labeled oligos that hybridized to the complementary sequences within the amplicon were visualized and quantified by microscopy image analysis. Leica M205 FA fluorescent stereoscope (Leica Microsystems, USA) and LAS-X-Core Software (3.7.4. version, LAS X Life Science, USA) were used. PLA results were quantified by Image J software (version 1.52a, NIH, USA) following user guide (https://imagej.nih.gov/ij/docs/guide/user-guide.pdf; https://www.unige.ch/medecine/bioimaging/files/1914/1208/6000/Quantification.pdf).

### Statistics

Data were analyzed by GraphPad Prism 5.02 software and are expressed as mean ± SD. At least two independent experiments were performed from eight IPF or Donors. About four to five PCLS from each IPF patient were pooled per each treatment group and PCLS from three IPF patients were used. Statistical significance of differences between two groups was assessed using the Mann–Whitney *U* test. For the statistical comparison of differences between ≥ 3 groups, one-way ANOVA was used. Significance level is indicated by *P < 0.05,**P < 0.01, and ***P < 0.001.

### Supplementary Information

Below is the link to the electronic supplementary material.

Supplementary file1 (DOCX 54 KB)

Supplementary file2 (TIFF 1258 KB)

Supplementary file3 (TIFF 3061 KB)

Supplementary file4 (TIFF 1975 KB)

Supplementary file5 (TIF 1543 KB)

Supplementary file6 (TIFF 2235 KB)

Supplementary file7 (TIFF 1092 KB)

Supplementary file8 (TIFF 3427 KB)

## Data Availability

All relevant data are within the manuscript and supplementary information.
