# Supplementary material for: PACS2–TRPV1 axis is required for ER–mitochondrial tethering during ER stress and lung fibrosis
Source: Cell Mol Life Sci. 2022 Feb 25;79(3):151. doi: 10.1007/s00018-022-04189-2 (PMC8881280; doi:10.1007/s00018-022-04189-2)
Supplement: Supplementary file 1 — Supplementary file1 (DOCX 54 KB) [file 18_2022_4189_MOESM1_ESM.docx]

**PACS2-TRPV1 axis is required for ER-mitochondrial tethering during ER stress**

**and lung fibrosis**

Jessica Knoell, Shashi Chillappagari, Lars Knudsen, Martina Korfei, Ruth Dartsch, Danny Jonigk, Mark P. Kuehnel, Konrad Hoetzenecker, Andreas Guenther, Poornima Mahavadi

*Corresponding author. Email: Poornima.mahavadi@innere.med.uni-giessen.de

**Supplemental Information**

**Supplementary methods**

**Gene Splicing by Overlap Extension (SOEing)**

As described by Horton et.al. [1] and Wang et al. [3], gene Splicing by Overlap Extension (SOEing) was performed to generate SPC^Δexon4^ by HighFidelity DNA Polymerase using specific primers (Metabion) as tabulated in Table S1.

Primers used for cloning EGFP-SPC^WT^ in pEGFP-C1 vector (pCMV promoter driven, originally from Clontech) with GFP tag to the N-terminal of the multiple cloning site are given in table 1 (ForSPC-hu & RevSPC-hu). Reference sequence for SPC^WT^ is Gene ID: 6440, *SFTPC*, *Homo sapiens*. An in-frame fusion protein was made through introduction of a HindIII site at the 5’ end and an EcoRI site at the 3’end for cloning into pEGFP-C1 generating pCMV-EGFP-hSPC^WT^plasmid. For generating pCMV-EGFP-hSPC^Δexon4^, deletion of exon 4 from pCMV-EGFP-hSPC^WT^was achieved by overlap extension PCR with a two round, four-primer technique. In step 1, exons 1-3 were amplified by single PCR using two primers: ForSPCint1-hu and RevSPCint1-hu. In step 2, exon 5 was amplified through the 3’ untranslated Poly A tail using a forward primer containing complete overlap with the 3’ end of the reverse primer used to amplify exon 3 (Primers: ForSPCint2-hu and RevSPCint2-hu in Table S1). The two overlapping primary products were fused by SOEing PCR using primers ForSPC-hu and RevSPC-hu. Following digestion with HindIII and EcoRI, the hSPC^Δexon4^ insert was subcloned into pEGFPC1 using standard protocols to generate pCMV-EGFP-hSPC^Δexon4^ plasmid. Several clones were generated and sequenced. Positive clones were identified after confirming the correct sequence for both plasmids.

**Stable transfection**

MEL188 cells were seeded in 6 well plates in full culture medium and allowed to grow overnight. GFP-SPC^WT^ Vector and GFP-SPC ^Δexon4^ were linearized with restriction enzyme ApaL I and purified with PCR purification Kit (Qiagen, Germany). Cells were transfected with linearized vectors with neomycin resistance gene. 24 h post-transfection medium was replaced with fresh medium containing 600 µg/mL geneticin (G418, Gibco, Germany) and allowed to grow for 10 days. Medium was replaced every second day. Stably transfected cells were identified by green fluorescence under light microscope and picked up with sterile pipette and transferred to 96 well plates. After 2 to 3 weeks of culturing cells in medium containing 600 µg/mL geneticin (replaced with fresh medium every third day), cells were transferred to 10 cm culture dishes until only neomycin resistant cells remained in culture. Several clones were picked and transfection efficiency and positive clones were harvested and analyzed for gene and protein expression.

**Co-Immunoprecipitation**

Cells were plated on 10 cm TC-Dish followed by treatment and/or transfection or left untreated. Co-IP was performed using anti-Myc antibody using protocols as we described before [2]. Briefly, fresh lysates in IP lysis buffer (Pierce) with protease inhibitor cocktail (Thermo Scientific, Germany) were transferred on to antibody immobilized magnetic beads (Dynabeads, Thermo Scientific) and incubated overnight at 4°C on a vertical rotator. Next day, supernatant was removed by placing eppi cups on magnetic stand, followed by washing the pellets. Pellets were then resuspended with 40 µL PBS. Samples were denaturated. For loading on SDS-Gel 5 µL for input and 5-20 µL for immunprecipitated sample were used

**Cytotoxicity Assay**

Cytotoxicity was measured using Lactate Dehydrogenase Activity Assay Kit (Sigma Aldrich, Germany) as per manufacturer’s instructions and as we described before. Briefly, cells were phenol-red free culture medium (Gibco, Germany) in a 96-well plate, induced or non-induced with dox followed by CPS treatments at indicated concentrations. For measurement cell death 50 µL of cell supernatants were mixed with 50 µL reaction mix (48 µL LDH Assay Buffer and 2 µL LDH Substrate Mix) and the absorbance at 450 nm (A450) was measured by microplate reader (Infinite® M200 Pro, Tecan Group Ltd, Switzerland). The raw data were analyzed for LDH activity using the following formula. For each sample two replicates were performed.

$$LDH activity [\frac{milliunits}{ml}]=\frac{{(A}_{450})final- {(A}_{450})inital}{{(T}_{final}- T_{inital})x V}$$

ΔA_450_ = amount of NADH generated by assay between T_inital_ and T_final_ [nmol]
T_inital_ -T_final_ = reaction time [min]

V = sample volume [mL]

**References**

1. Horton R M (1995) PCR-mediated recombination and mutagenesis. SOEing together tailor-made genes. Mol Biotechnol 3(2): 93–99

2. Kesireddy V S, Chillappagari S, Ahuja S et al (2019) Susceptibility of microtubule-associated protein 1 light chain 3β (MAP1LC3B/LC3B) knockout mice to lung injury and fibrosis. FASEB J 33(11): 12392–12408

3. Wang W-J, Mulugeta S, Russo S J et al (2003) Deletion of exon 4 from human surfactant protein C results in aggresome formation and generation of a dominant negative. Journal of Cell Science 116(Pt 4): 683–692

**Figure S1. Induction of apoptosis in cells overexpressing *Chop***

1. Immunoblot analysis of cleaved Parp1 in total cell lysates of MLE12 cells untreated or treated with dox for Chop expression for 12 and 24 hours.
2. Relative protein amounts were normalized to Gapdh and their level in –dox cells was set to one.

**Figure S2. Doxycycline treatment alone has no effect on ER-mitochondrial contacts**

A. Immuno blot analysis of Pacs2 in total cell lysates of healthy MLE12 cells with or without 1 µg/mL doxycycline.

B. Proximity ligation assay of MLE12 cells – or + dox treatment with antibodies against Calnexin and VDAC1 followed by fluorescence microscopy, scale bar = 60 µm.

**Figure S3. Knock down of Pacs2 in MLE12 cells**

Control MLE12 cells were transfected wither with non targeting (NT) siRNA or with Pacs2 siRNA for 48 hours followed by A. immunoblot for Pacs2 and B. its quantification to check for the specificity of Pacs2 antibody.

**Figure S4. cIAP proteins are not affected upon Chop overexpression**

A. Immuno blot analysis of cIAP proteins in total cell lysates of MLE12 cells untreated or treated with dox for Chop expression for 12 and 24 hours

B. Relative protein amounts were normalized to Gapdh and their level in –dox cells was set as one.

**Figure S5. Pacs2 directly interacts with Trpv1**

A. MLE12 cells overexpressing *Chop* were either transfected with empty-Myc or Myc-PACS2 followed by treatment with or without dox. Co-IP using anti-Myc and immune blots for Myc, Pacs2 and Trpv1 are shown.

**Figure S6. Cytotoxicity assay following CPS treatment**

A. Quantification of LDH assay of MLE12 12 cells overexpressing Chop followed by treatments with different CPS concentrations (5 µM,10 µM, 25 µM). Relative LDH activity were normalized to basal level. Analysis from n=3 independent experiments are shown. p values were not significant between the groups.

**Figure S7. Gene Splicing by Overlap Extension for generation SPC^Δexon4^.**

A. Schematic representation of Gene Splicing by Overlap Extension PCR reaction (SOEing PCR) resulting in depletion of Exon 4 of SPC gene.

B. Agarose gel picture showing Product 1 (324 bp) and Product 2 (159 bp) and the fused product (483 bp) resulting in *SPC^Δexon4^*.

Supplementary table 1.

| **Name** | **Primer Sequence (5’**🡪**3’)** |
| --- | --- |
| ForSPC-hu | TATTATAAAGCTTATGGATGTGGGCAGCAAAGAGGTCCTG |
| RevSPC-hu | TATTATGAATTCCTAGATGTAGTAGAGCGGCACCTCGCCAC |
| ForSPCint1-hu | TACAAGTCCGGAATGGATGTGGGCAGCAAAGAGGTCCTG |
| RevSPCint1-hu | CTGCTGGTAGTCATACACCACGAGGCC |
| ForSPCint2-hu | GGCCTCGTGGTGTATGACTACCAGCAGATGGAATGCTCTCTGCAGGCCAAGCCC |
| RevSPCint2-hu | TATTATCTCGAGCTAGATGTAGTAGAGCGGCACCTCGCCAC |

**Table S1.** List of primers used for cloning. Underlined bases in each primer represent restriction sites used for cloning.
