# Supplementary figures and images for: PACS2–TRPV1 axis is required for ER–mitochondrial tethering during ER stress and lung fibrosis
Source: Cell Mol Life Sci. 2022 Feb 25;79(3):151. doi: 10.1007/s00018-022-04189-2 (PMC8881280; doi:10.1007/s00018-022-04189-2)

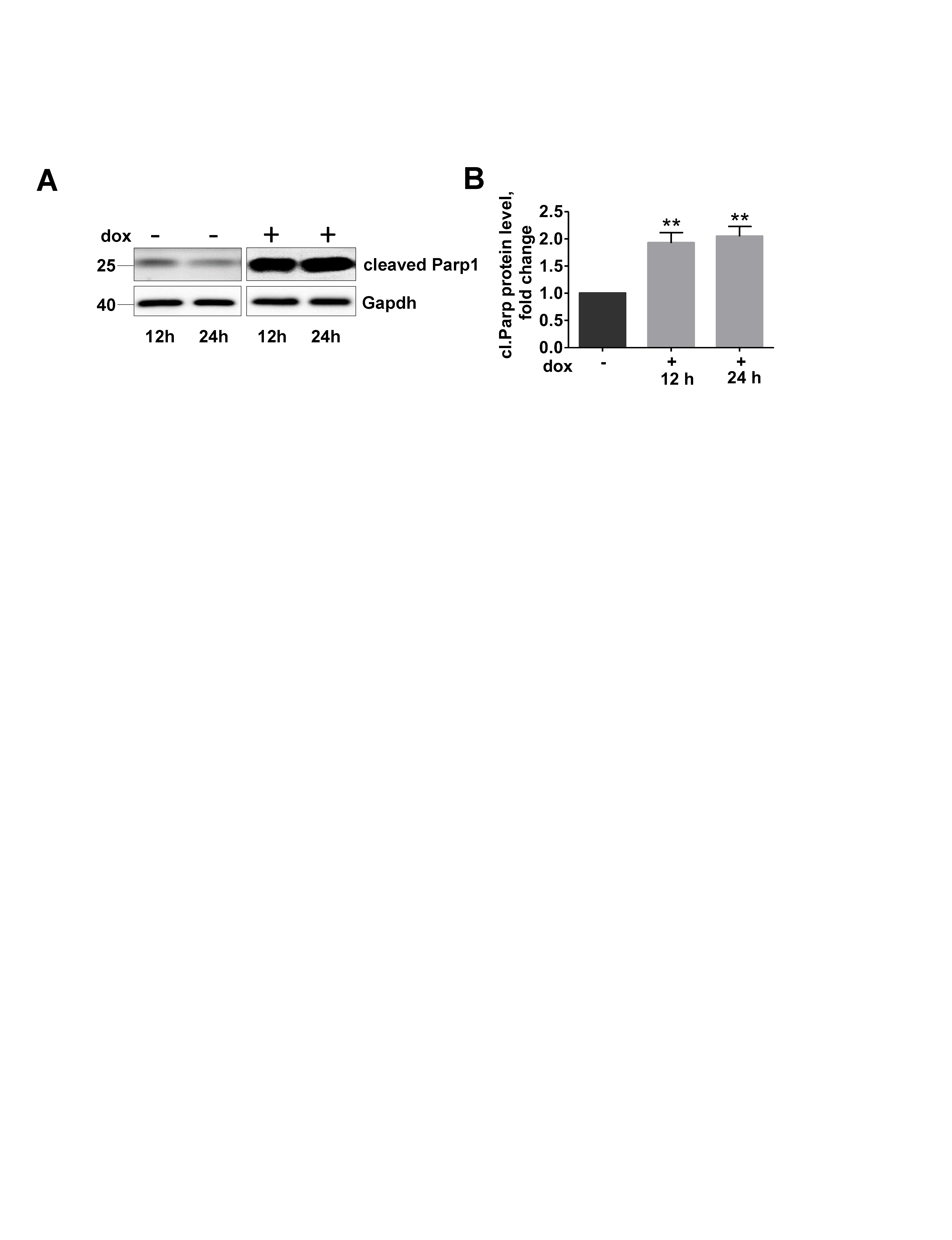

Supplement: Supplementary file 2 — Supplementary file2 (TIFF 1258 KB) [file 18_2022_4189_MOESM2_ESM.tiff]

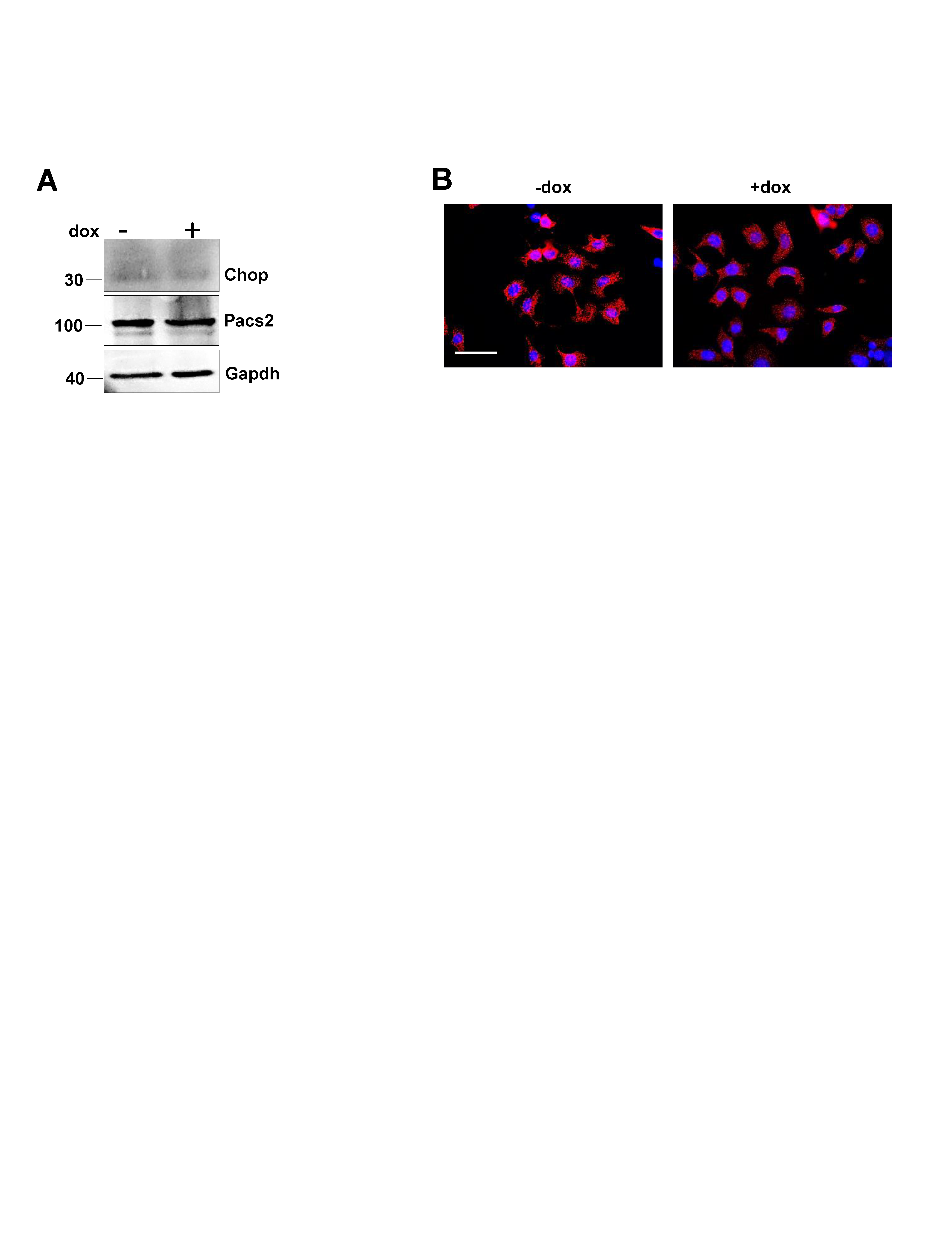

Supplement: Supplementary file 3 — Supplementary file3 (TIFF 3061 KB) [file 18_2022_4189_MOESM3_ESM.tiff]

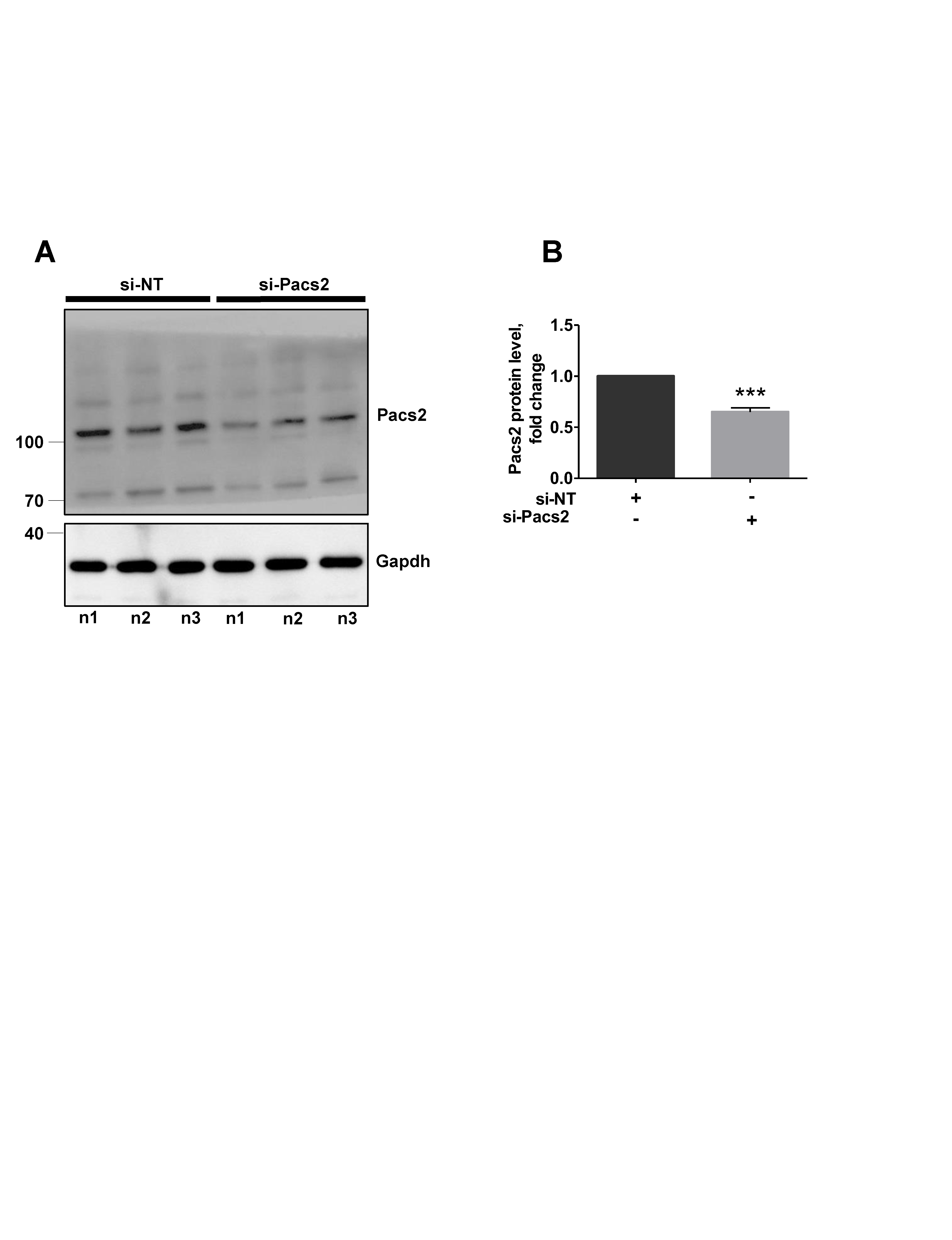

Supplement: Supplementary file 4 — Supplementary file4 (TIFF 1975 KB) [file 18_2022_4189_MOESM4_ESM.tiff]

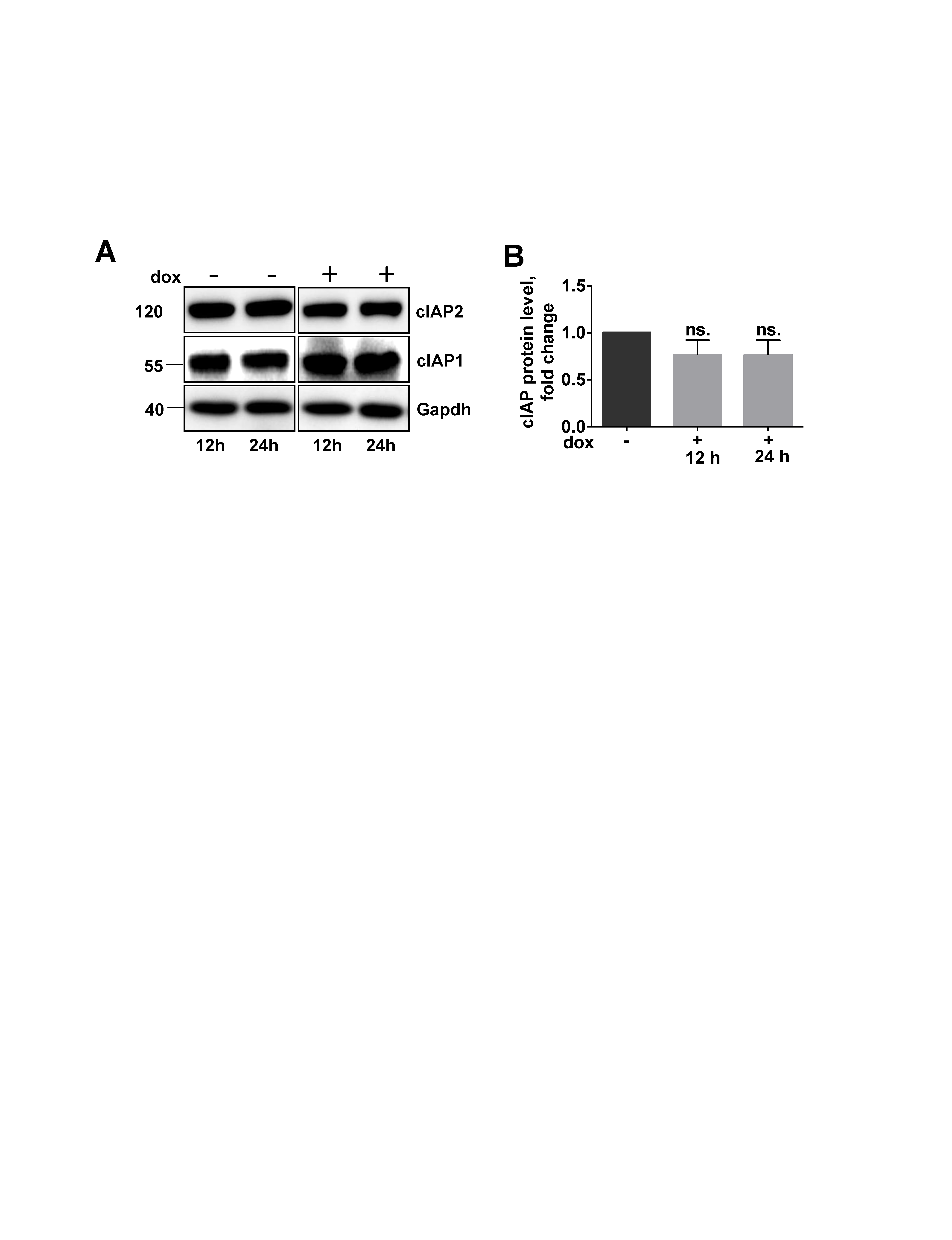

Supplement: Supplementary file 5 — Supplementary file5 (TIF 1543 KB) [file 18_2022_4189_MOESM5_ESM.tif]

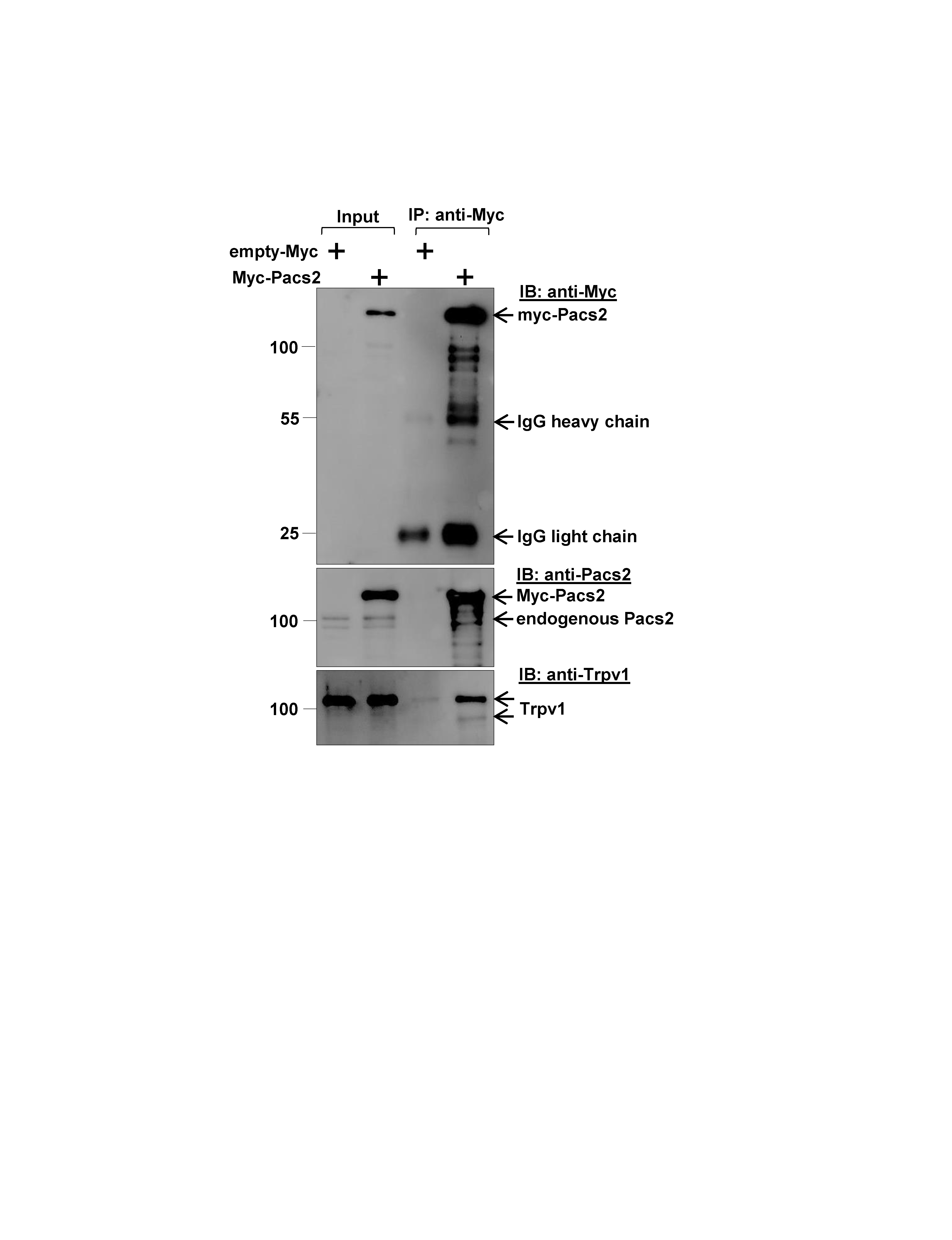

Supplement: Supplementary file 6 — Supplementary file6 (TIFF 2235 KB) [file 18_2022_4189_MOESM6_ESM.tiff]

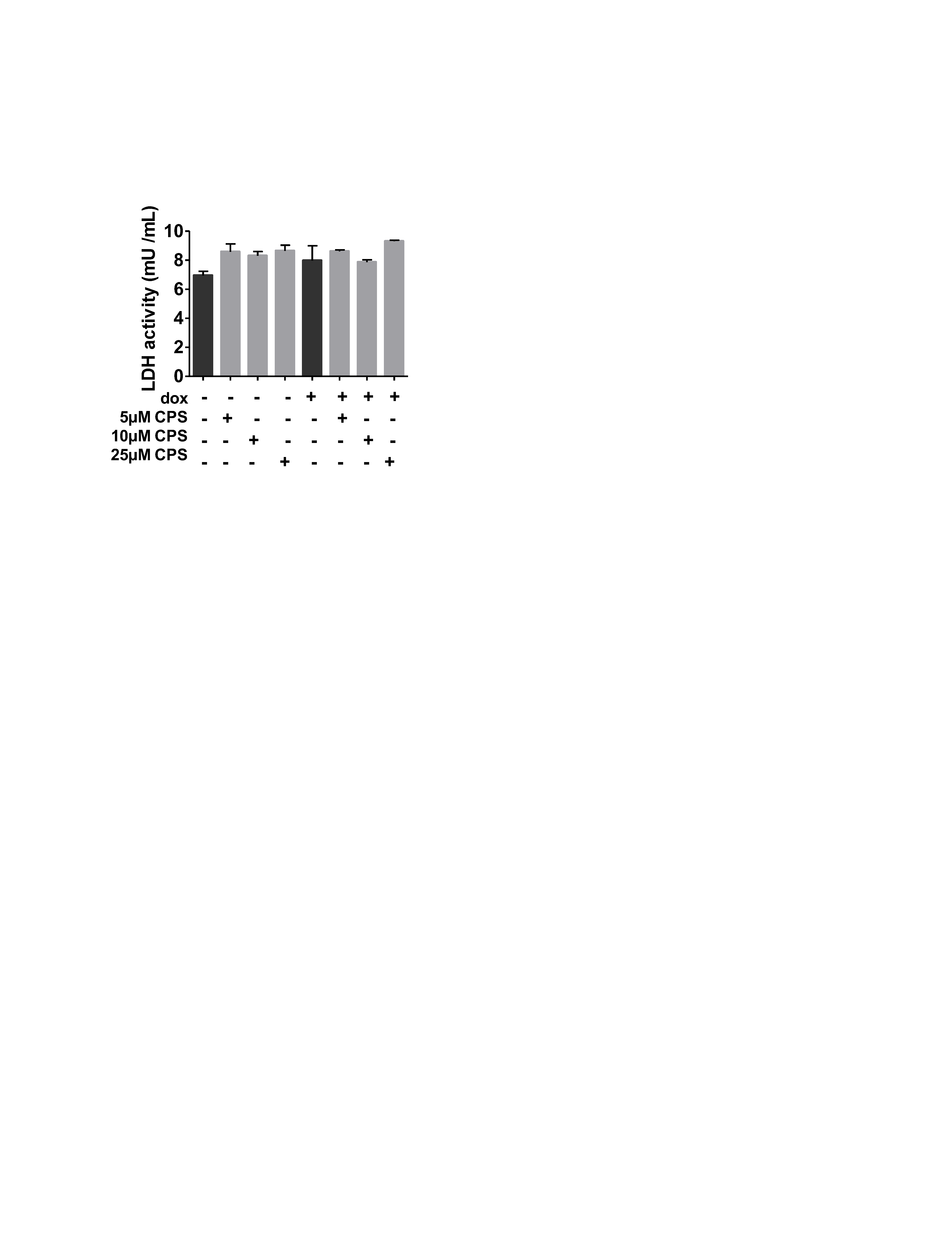

Supplement: Supplementary file 7 — Supplementary file7 (TIFF 1092 KB) [file 18_2022_4189_MOESM7_ESM.tiff]

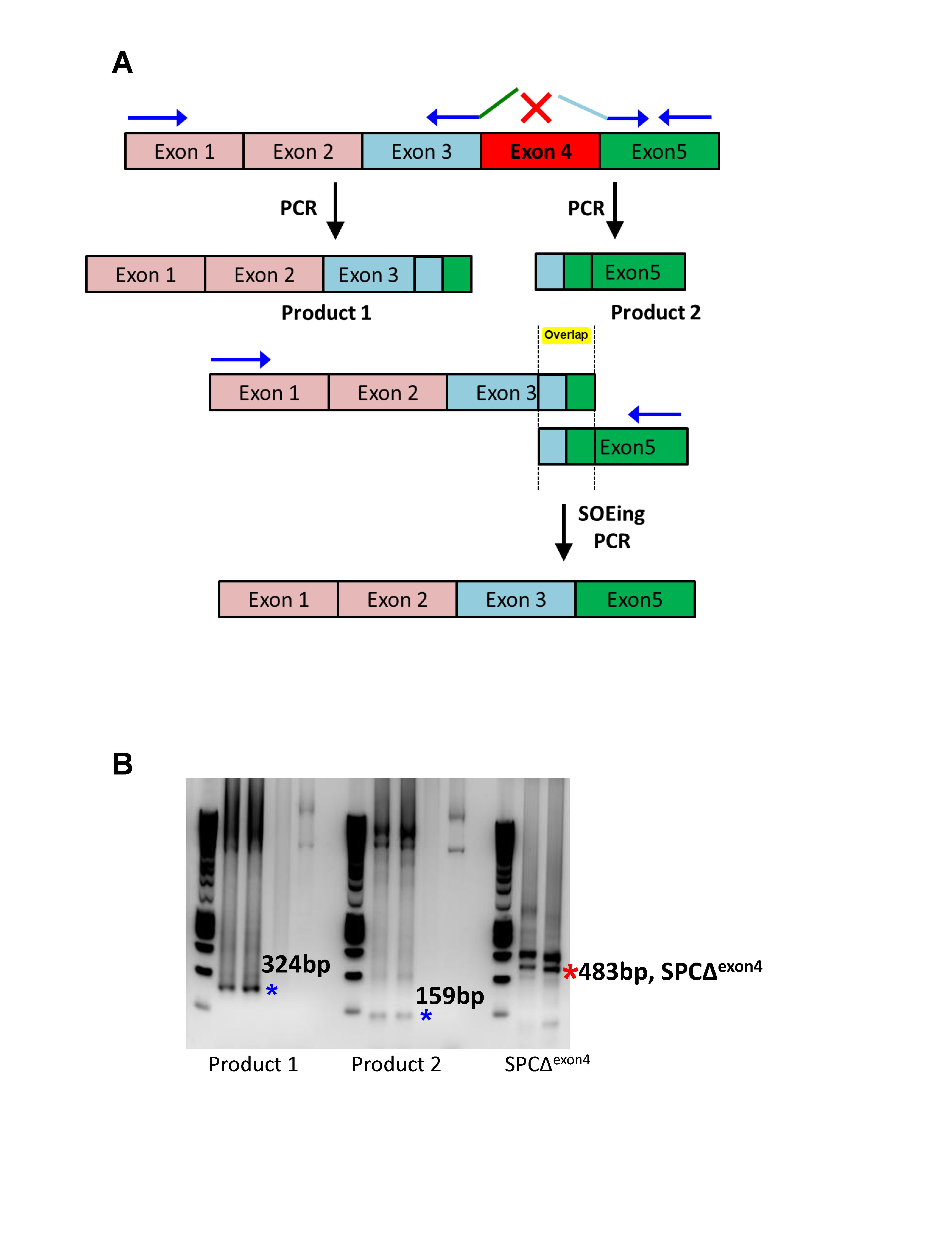

Supplement: Supplementary file 8 — Supplementary file8 (TIFF 3427 KB) [file 18_2022_4189_MOESM8_ESM.tiff]
